# Layered vulnerability and researchers’ responsibilities: learning from research involving Kenyan adolescents living with perinatal HIV infection

**DOI:** 10.1186/s12910-023-00972-3

**Published:** 2024-02-20

**Authors:** Mary Kimani, Sassy Molyneux, Anderson Charo, Scholastica M. Zakayo, Gladys Sanga, Rita Njeru, Alun Davies, Maureen Kelley, Amina Abubakar, Vicki Marsh

**Affiliations:** 1 Kenya Medical Research Institute Wellcome Trust Research Programme, Kilifi, Kenya; 2 Health Systems Collaborative, Centre for Global Health Research, Nuffield Department of Medicine, University of Oxford, Oxford, UK; 3 Ethox Centre, Nuffield Department of Population Health, University of Oxford, Oxford, UK; 4 Aga Khan University, Institute for Human Development, Nairobi, Kenya; 5 Centre for Global Health Research, Nuffield Department of Medicine, University of Oxford, Peter Medawar Building for Pathogen Biology, 3 South Parks Road, Oxford, OX13SY UK

**Keywords:** Africa, Global health research, Research ethics, Empirical ethics research, Adolescent health, HIV/AIDS, Vulnerability, Resilience, Ancillary care, Researchers’ responsibilities

## Abstract

**Background:**

Carefully planned research is critical to developing policies and interventions that counter physical, psychological and social challenges faced by young people living with HIV/AIDS, without increasing burdens. Such studies, however, must navigate a ‘vulnerability paradox’, since including potentially vulnerable groups also risks unintentionally worsening their situation. Through embedded social science research, linked to a cohort study involving Adolescents Living with HIV/AIDS (ALH) in Kenya, we develop an account of researchers’ responsibilities towards young people, incorporating concepts of vulnerability, resilience, and agency as ‘interacting layers’.

**Methods:**

Using a qualitative, iterative approach across three linked data collection phases including interviews, group discussions, observations and a participatory workshop, we explored stakeholders’ perspectives on vulnerability and resilience of young people living with HIV/AIDS, in relation to home and community, school, health care and health research participation. A total of 62 policy, provider, research, and community-based stakeholders were involved, including 27 ALH participating in a longitudinal cohort study. Data analysis drew on a Framework Analysis approach; ethical analysis adapts Luna’s layered account of vulnerability.

**Results:**

ALH experienced forms of vulnerability and resilience in their daily lives in which socioeconomic context, institutional policies, organisational systems and interpersonal relations were key, interrelated influences. Anticipated and experienced forms of stigma and discrimination in schools, health clinics and communities were linked to actions undermining ART adherence, worsening physical and mental health, and poor educational outcomes, indicating cascading forms of vulnerability, resulting in worsened vulnerabilities. Positive inputs within and across sectors could build resilience, improve outcomes, and support positive research experiences.

**Conclusions:**

The most serious forms of vulnerability faced by ALH in the cohort study were related to structural, inter-sectoral influences, unrelated to study participation and underscored by constraints to their agency. Vulnerabilities, including cascading forms, were potentially responsive to policy-based and interpersonal actions. Stakeholder engagement supported cohort design and implementation, building privacy, stakeholder understanding, interpersonal relations and ancillary care policies. Structural forms of vulnerability underscore researchers’ responsibilities to work within multi-sectoral partnerships to plan and implement studies involving ALH, share findings in a timely way and contribute to policies addressing known causes of vulnerabilities.

**Supplementary Information:**

The online version contains supplementary material available at 10.1186/s12910-023-00972-3.

## Background

The rising rates of horizontal HIV infection and the improved survival of children with perinatal HIV infection have led to a high number of adolescents living with HIV (ALH) [1–3]. For this group, adherence to antiretroviral medicines is lifesaving but often problematic, including through experiences of stigma and discrimination [1, 4]. For many ALH, the fears and harms of stigma and discrimination remain common, manifested in three ways: anticipating that actions or inactions may be stigmatising; experiencing or perceiving other’s actions as stigmatising; and internalising others’ negative views about oneself [2, 4–7]. These challenges are particularly important given the formative nature of the adolescent period, when life experiences shape individual capabilities and human potential, as well as physical, emotional, cognitive and behavioural development [8]. To address individual, family and societal-level challenges, evidence-based programmes of prevention, control and care are critical and require an understanding of how ALH can be involved fairly in needed research.

The dilemma of how to involve ALH in much-needed research exemplifies a well described ‘vulnerability paradox’ in research ethics, balancing the need for evidence-based policies and interventions for groups seen as vulnerable with concerns that their involvement in research will deepen underlying causes of vulnerability [9, 10]. The concept of vulnerability in research ethics guidance has evolved over the past decade, moving from population-based accounts (for example, ‘prisoners’ and ‘children’) towards a more targeted and contextual evaluation of individual experiences within groups, identifying common, specified risks [9, 10]. Thus, while ALH viewed as a ‘population’ may need extra protections related to their age and stage of development, these considerations should be placed in context. Important contextual elements include questions around emerging competence to make decisions, a need for legal protection as minors and related concerns around protecting young people’s best interests while respecting their emerging agency or ability to choose and take action [9, 11]. Further, within this sub-population of young people, there should be special attention to patterns of susceptibility to specific risks, such as stigma related to living with and treating HIV. Additionally, we know that for many individual young people, health, socioeconomic and cultural influences at individual, family, community, and wider structural levels underpin patterns of vulnerability and resilience [12–14]. Recognising this more complicated picture, definitions of vulnerability in research have increasingly accounted for individual and context-specific influences, informing more precise measures of what Hurst defends as a central criterion of ‘an increased likelihood of incurring additional or greater wrong’ [15].

In this paper, we draw on Luna’s (2009) account of vulnerability in the research ethics literature, identifying the need for a finely granular understanding of contextual influences on an individual’s potential for vulnerability and the way that these influences may act as overlapping and interrelated ‘layers’, shifting across time and space [16]. The use of ‘layers’ as a metaphor acts as a counter to forms of labelling or taxonomy that may be too rigid to capture the fluidity and interconnected nature of influences on vulnerability in reality. Luna (2019) further elaborates an approach to understanding researchers’ responsibilities through assessing levels of importance and urgency around specific forms of vulnerability, offering guidance to Ethics Review Committees (ERCs) and researchers [17]. Through this analysis, Luna highlights a particularly important risk of the emergence of vicious cycles, described as a vulnerability cascade, in which potentially vulnerable individuals take actions that worsen their situation, often reflecting forms of agency constrained, or bounded, by sociocultural and economic influences [18, 19].

Luna’s account is a conceptual framework and as such requires further empirical understanding of how complex, layered sources of vulnerability in daily living are brought to the research experience and, in turn, might be balanced with other important values like agency and resilience to inform researcher responsibilities in real world research [20]. We designed an empirical ethics study to explore these responsibilities, based on a research partnership between social and neurobehavioural scientists at the KEMRI Wellcome Trust Research Programme (KWTRP) in coastal Kenya [21]. The empirical ethics study was nested within an ongoing longitudinal observational cohort study aiming to assess the impact of perinatal HIV infection on cognitive, educational, mental and social outcomes for ALH in Kilifi County (Adolescent Health Outcomes Study or AHOS) [22]. Further, our empirical ethics research formed part of a multi-country collaborative project exploring Resilience and Empowerment in Maternal and Child Health Research (REACH). Additional file 1 (AF1), titled ‘A summary of AHOS.pdf’, provides a description of AHOS, including the study aims, methods and approaches taken to addressing anticipated ethical issues, including through ancillary care planning.

Across the paper, we draw on Luna’s account (2009; 2019) to share a nuanced understanding of the specific and multi-layered nature of vulnerability and resilience for young people living with HIV, including everyday experiences in school, in seeking health care, in life at home, in the community and during participation in AHOS. Our findings explore the relationship between vulnerabilities across everyday life and research participation, with wider implications for research policy and practice, underlining the importance of institutional context and collaborative partnerships [17].

## Study methods

### Study setting & population

Our study was based at the Kenya Medical Research Institute (KEMRI) Wellcome Trust Research Programme (KWTRP) in Kenya, a long standing international collaborative research programme with its main hub in Kilifi County, a largely rural area of coastal Kenya [21]. Studies at KWTRP in Kilifi, including AHOS, often involve participants from surrounding areas, with research governance drawing on a collaborative partnership with the Kilifi County Health Team. The main economic activities across the county include petty trade, subsistence farming and fishing alongside emerging urban development, with rates of multidimensional poverty assessed at 35.6% [23].

### The empirical ethics study: approach and methods

**Fig. 1:**
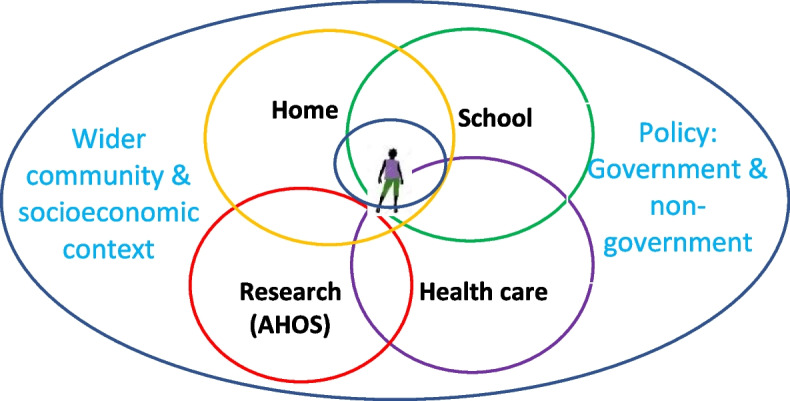
Planned research foci for the empirical ethics study

As an empirical ethics study, we used qualitative methods to explore vulnerability, resilience and agency in everyday life for ALH who joined AHOS, including influences from research participation. ALH in AHOS were largely school-going, attending day or boarding schools. As shown in Fig. 1, our study focused on three main domains in young people’s lives: the school environment, HIV Comprehensive Care Clinics (CCCs), and home and community. Research participation in AHOS is treated as cross-cutting, as are wider structural and policy influences [24].

### Data collection, management and analysis

We collected data across three main research phases, with on-going analysis. To counter any potential for our research to exacerbate existing vulnerabilities and increase the potential for participation to be empowering for young people involved, we began by working with key stakeholders and least vulnerable ALH to learn about perceptions of vulnerability, resilience and sources of support before involving a wider group, primarily through a participatory workshop as described below. Table 1 gives a summary of study methods, participants and numbers involved.

**Table 1:** Study participants and data collection activities

**Types of participants**	**Data collection activity (number)**	**Number of participants**
*Health related:* Clinical officers (2), nurse (1), Health policy and coordination of HIV activities (3)	In depth Interview (IDI) (6)	6
*School related:* School head (1), school matrons (2), teacher (1), teachers living with HIV (3)	IDI (4) Group interview (1)	7
*Community related:* Community health volunteers (2), mentor mothers (3), children officer (1), NGO project officer (1)	IDI (7)	7
*Family related:* Parents/caregivers	Focus group discussions (2) Group interview (1)	13
*AHOS related:* Recruitment staff (1), community liaison officer (1)	IDI (2)	2
*Adolescents:* Adolescents living with HIV	Group interview before workshop (1)	3
	Focus group discussions: Within (3) & after (2) workshop	24
Total number of participants		62

Phase 1 included 15 in-depth interviews with key informants and two group interviews, one with ALH and the other with their main caregivers. In-depth interviews included policy and provider stakeholders working with ALH in the community, schools, CCCs and as part of AHOS, identified purposively in relation to formal and informal roles, including through a snowballing process. In this way, we asked policy and provider stakeholders to recommend others with relevant experience to involve, and continued to identify potential participants in this way as an going process throughout the study [25]. In the health sector, we interviewed policy, management and provider stakeholders, working at County and facility levels, including from three CCCs. These clinics were selected purposively to include the main urban CCC in this setting and two facilities in rural (*n* = 2) settings that were used by a majority of AHOS participants. We invited teaching and pastoral care staff from six purposively selected schools in Kilifi County, reflecting diversity in setting (urban vs. rural), pupil size, educational level (five primary and one secondary school) and status as day schools or those offering both day and boarding facilities. Across all sectors, interviews focused on identifying every day and research-related vulnerabilities and resilience for ALH and recommendations around research design and conduct. In this early phase, for focus group discussions (FGDs) with young people and their caregivers (as separate groups), we purposively selected ALH acting as ‘HIV champions’ in local CCCs, as a group seen by policy and provider stakeholders as least vulnerable and most likely to be able to reflect on their own and their contemporaries’ situations. FGDs in Phases 1 and 3 explored understanding and experiences of participating in AHOS, probing around spontaneously shared accounts of everyday vulnerability.

**Fig. 2:**
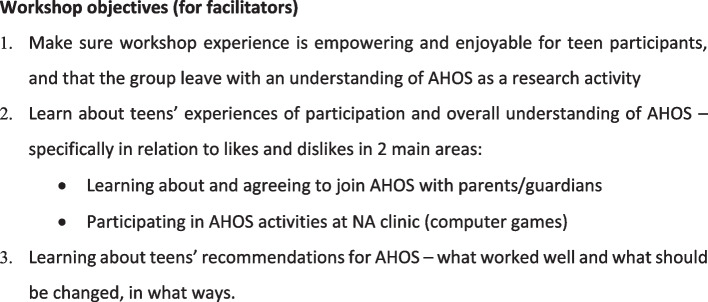
Workshop objectives in Phase 2

**Fig. 3:**
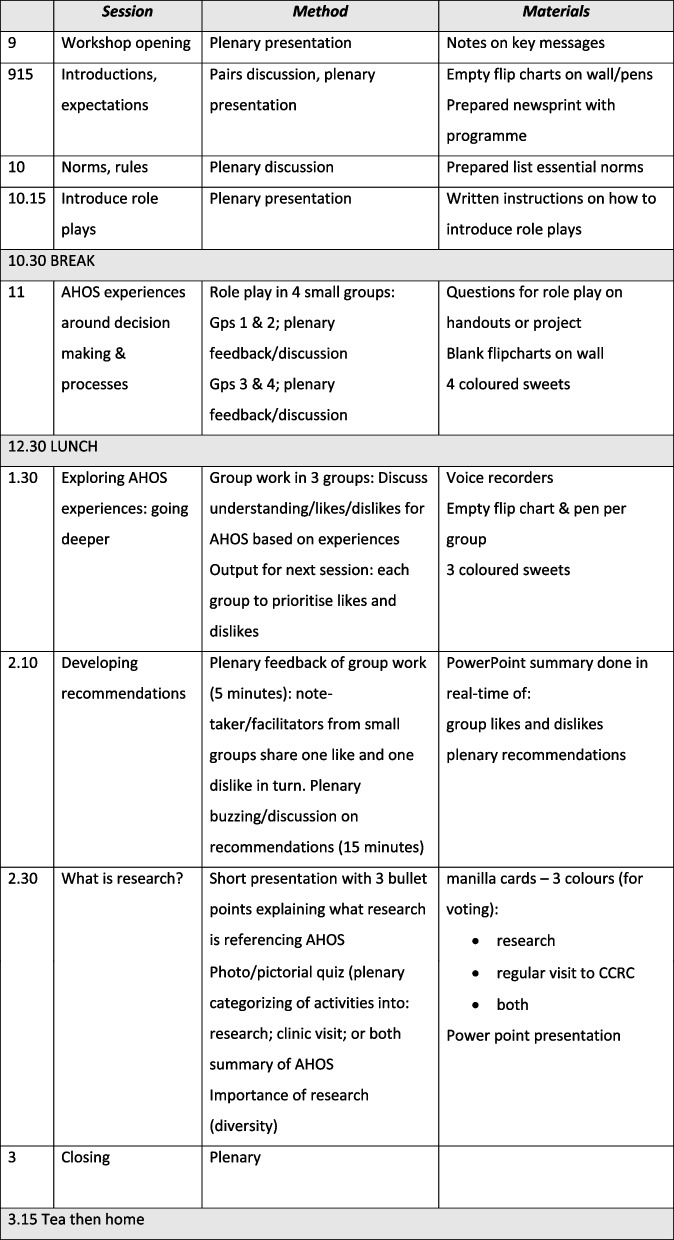
Workshop programme in Phase 2

Phase 2 included a one-day consultative workshop, drawing on Phase 1 findings and involved 24 ALH who were AHOS participants. Purposive sampling aimed to maximize diversity of social and demographic characteristics (gender, age, geographic distribution, type of school attended). We held the workshop in a relaxed local hotel environment during a school holiday and included meals. Activities included group discussions and role-plays around experiences of AHOS participation in the morning, followed up in FGDs in the afternoon (*n* = 3) involving the 24 ALH. The workshop ended with a plenary discussion on the nature and aims of health research, using visual aids and using AHOS as an example, to build general understanding of research and address emerging issues. Workshop objectives are given in Fig. 2 and the workshop programme in Fig. 3.

Phase 3 involved four in-depth interviews and one group interview with key informants, two FGDs with workshop attendees and two FGDs with main caregivers, to clarify and take forwards findings from earlier phases.

In Phases 1 and 3, group discussions/interviews with ALH and caregivers lasted 40–90 min, with breaks for those involving ALH. Interviews with key stakeholders lasted 50–110 min. The data collection tools were developed as part of this study and are included in Additional file 2, titled ‘Data collection tools.pdf’, showing the last versions of each tool used. In practice, all tools evolved to build on learning over time during the data collection period. Data collection used Swahili, local (Mijikenda) or English languages, following participants’ preferences.

Interviews were audio recorded, transcribed verbatim and translated into English where necessary. Data analysis processes were ongoing, iterative and informed by a Framework Analysis approach, using NVivo 10 software [25]. Analysis involved the following processes: i) immersion in the data during collection; ii) the development, use and adaptation of a priori and emergent codes from an initial sample of rich transcripts by two researchers (MN and VM); iii) the development of analysis charts drawing on coded material; and iv) data interpretation using the literature on vulnerability and resilience in ALH and global health research ethics (MN, AC, VM, SM). These processes drew on repeated readings of and team discussions around the data, both during and following data collection, as an iterative process.

Reflecting the iterative nature of the enquiry, the coding framework evolved over the data collection and analysis periods to include early and emerging areas. Table 2 describes the main foci of the analysis, with areas in standard font reflecting earlier codes, and those in italics highlighting later codes, as this process evolved. The broad theme on vulnerability and resilience was particularly cross-cutting and interpretive in nature, and was developed over time while keeping data as intact as possible, to illustrate the interrelated ways in which outcomes and their influences were experienced and described over time.

**Table 2:** Coding framework for analysis (early codes in standard font; later codes in italic)

Main themes	Codes
1. Forms/impacts of challenges and support at INDIVIDUAL level	• Physical health • Individual attitudes & knowledge around HIV/AIDS • Individual risk behaviours
2. Forms/impacts of challenges and support AT HOME	• Economic issues • Family attitudes & relations (inc external stigma, family taking drugs openly together), including guardianship/orphans • Friends/peer group at home/community • *Supporting adherence*
3. Forms/impacts of challenges & support at CCC/MOH	• Policy level influences • CCC staff attitudes and communication • Accessing CCCs • Perceptions of quality of services • Waiting/timing of clinics • Youth friendly services • Recommendations for clinics • External agencies supporting CCCs • *Choosing/changing clinics* • *Travelling long distances to clinic*
4. Forms/impact of challenges & support at school	• Policies impacting ALHIV at school • Choosing types of school • Staff attitudes & actions • *Stories of lack of privacy to take drugs, and impact* • *Fear of unwanted disclosure including boarding schools* • *Attending CCC/clinics from school* • *Impact of HIV / challenges and support on schooling*
5. Policy level influences (areas)	• Pregnancy support/adherence in pregnancy/other • Disclosure in family/for child • Family counselling/ emotional support/practical support/ mentor mothers • Messaging to ALHIV e.g. contraception/sex • HW training • *Friends/peer group/champions support/boot camps* *Agencies working together*
6. Research participation	• Making decisions about participation • Reasons for joining/staying • Understanding/remembering AHOS • Challenges for ALH in attending AHOS clinics • Participating over time • Recommendations for research (pos & neg) • Community perceptions research/KEMRI/AHOS • *Teen-parent dynamics* • *Being accompanied* • *Ensuring privacy* • *Supportive staff attitudes* • *Enjoying research activities* • *Ethical challenges and strengths around informed consent processes*
7. Vulnerability & resilience: Illustrative accounts and ongoing impacts – drawing on findings on ‘support’ and ‘challenges’ across earlier themes	• *How and why vulnerability/resilience emerge across the data through cascades/processes* • *Ways in which agency can be constrained/bounded, relationship to vulnerability cascades* • *Role of supportive individuals in promoting resilience/countering vulnerability*

### Ethical considerations

We planned this study with high awareness of existing vulnerabilities for ALH and their families, and particular concern to protect young people’s privacy and respond appropriately to challenges uncovered during our research. In so doing, we drew on ancillary care plans developed for the main AHOS cohort through collaborative partnerships with County Health and Social Care departments, described in Additional file 1. Similarly, our consent processes drew on approaches developed for AHOS, including that a known and trusted individual was responsible for communicating about the study with the young people and caregivers involved, also described in Additional file 1. Adult participants gave written informed consent before involvement. Young people who were minors (under 18 years) gave verbal informed assent alongside adult caregivers’ informed consent, including for participation in groups involving other ALH who were AHOS participants. Heads of the Kilifi County Health and Education Departments and the Kilifi National AIDS and Sexually Transmitted Diseases Programme (NASCOP) supported study planning and conduct, and we communicated findings with these groups. The Kilifi County Research Governance office, the KEMRI Scientific and Ethics Review Unit (KEMRI/SERU/CGMR-C/084/3454) and Oxford University Tropical Medicine Research Ethics Committee (OXTREC 14–17) gave prior approval for the study.

## Results

Following the framework that guided data collection, shown in Fig. 1, we describe our findings on influences on vulnerability and resilience in relation to young people’s experiences in the community and home, in school, in attending HIV/AIDS Comprehensive Care Clinics (CCCs) and by participating in AHOS. Since vulnerability and resilience presented as “two sides of the coin” in young people’s daily lives, we present our findings with dual consideration of both aspects to better understand a young person's situation in context and to better target complex, supportive interventions. Across these four sections, we identify influences related to policies and organisational systems, and to interpersonal relations, within these environments.

### Community and home: influences on ALH vulnerability and resilience

Many county-level HIV policy makers and community-based providers saw community-wide stigma and discrimination towards people living with HIV as becoming less marked over time. At the same time, negative attitudes were reportedly common across the community, including widespread inaccurate understandings of perinatal HIV that underpinned discriminatory behaviour, such as accusations of irresponsible sexual behaviour, as has been widely reported in different settings [26, 27]. Key informants had experience of families feeling forced to move home, sometimes repeatedly, when they suspected disclosure and stigma, generating issues for continuity of care at CCCs and worsening outcomes. While poverty is common across rural areas of Kilifi county, ALH caregivers are particularly impacted, generating difficulties in accessing education, maintaining adequate nutrition and growth (core to fears and experiences of stigma), paying fares to attend CCC and being able to afford a radio, watch or phone to support timing of ART doses [27, 28]. Schooling could be impacted where girls were unable to afford sanitary towels and where hunger affected concentration in class. ALHs’ responses to these challenges risked deepening their vulnerability, for example, in refusing to take ARTs because they did not have food, turning to commercial sex work or leaving school to look for paid work. Other reported responses reflected less constrained (but still non-ideal) forms of agency, for example, getting up very early to walk to school in the absence of fares [29]. Across these accounts, economic challenges in the community and home environment acted as intersectional influences on ALHs’ wellbeing and ability to cope with the multiple, often severe, challenges faced in everyday life, as we describe across the following sections.

Considerable diversity emerged around accounts of the types and levels of practical and emotional support available to ALH at home, including medicine taking, as has been shown elsewhere, including in Kenya [30–33]. While some caregivers assumed that ALH could ‘look after themselves’ or ‘harassed’ those who forgot to take their ARTs, other families took their ARTS together. In one family, a young person and the parent took turns to remind each other to take their medication. We later discuss the privacy challenges faced by ALH in seeking time out of school to attend HIV/AIDS Comprehensive Care Clinics; caregivers could provide important support in this respect:

*My child doesn’t even ask for a leave out [from school] if it’s her time to come for the drugs, I usually call the teacher and inform him that my child had gone to the hospital and will come to school late. (Caregivers FGD04)*

Encouraging ART adherence was particularly challenging if ALH were not aware of their status, reportedly common in low-resource settings. Some caregivers had insisted that CCC staff take responsibility for disclosing their child’s status, contrary to national policy [34, 35]. Discriminatory attitudes were particularly described when affected young people were in the care of guardians or relatives outside their immediate family. It is notable that just over half of ALH participating in AHOS were orphans.^1^ Instances of prejudice and discrimination described included ALH being forced to keep their utensils separate from those of the wider household or sleep separate from other children in the home; caregivers selling food given for an ALH by local NGOs; and a ‘sponsor’ refusing to let an ALH placed in her care attend CCC. Elderly caregivers often struggled to provide an adequate diet given economic constraints, and faced difficulties in understanding and remembering ART regimes.

*…you know most of the kids we have here are taken care of by care givers, the first parents is not there…most of their care givers are grandmothers and they have their own challenges. Maybe they don’t even have food…also another issue, their grandmother doesn’t have the knowledge about medication … [but] once you teach them and they understand, they don’t deviate from that, they will do the right thing (Healthcare provider IDI20)*

Across the county, we learned that government departments and NGOs provide important support to communities and families living with HIV, including through CCCs. While operating on fixed funding cycles, community-based NGO staff had particularly valued roles in acting as mentor mothers to families living with HIV/AIDS. This group also distributed designated support to families in NGO programs, ran motivational ‘boot camps’ for ALH, during which they would be encouraged to take their ARTs openly together, and set up support groups to help parents disclose their children’s HIV status – a process seen as critical to ALH acceptance and wellbeing. Many such staff formed close and supporting relationships with the families and young people in their care, including supporting families in greatest need from their own pockets.

### School environments: influences on ALH vulnerability and resilience

Since AHOS participants were generally school going, those invited to join our study were primary or secondary school students in day, boarding or mixed facilities. The boarding school environment could be particularly challenging for ALH since, as shown earlier, family support was an important influence on young people’s ability to thrive outside the home. The primary challenge for ALH in schools centered on efforts to maintain privacy in relation to their HIV status given actual and perceived risks of stigmatisation by students and staff, as reported from other parts of Kenya and Sub-Saharan Africa more widely [36–40]. At the same time, staff could be a potential source of support to ALH, particularly members of the Kenya Network for Positive Teachers (KENEPOTE), a network set up within the Teachers Service Commission of Kenya in 2004 to support and empower this group [41]. Across the following paragraphs, we first describe challenges related to organisational policies in schools, followed by influences on vulnerability and resilience from interpersonal relations.

Systems in place for managing routine medications and regulating student movements out of school, particularly in boarding schools, presented the main privacy risks for ALH and challenges to ART adherence. As noted elsewhere, particular challenges related to storage and access to ARTs in schools and securing permission to leave school to attend regular HIV/AIDS CCC appointments [42]. Notably, policies that required advance permission to be absent from school for health or other reasons, or to show a medical note to explain an absence, while reasonable at face value, generated important privacy risks for ALH. Young people described taking a series of strategies to counter privacy risks, such as missing ART doses, taking leave-outs without permission and accepting unfair punishments:

*I just went to [started] that school just the other day. Better my former school where there was a teacher who also took drugs [ARTs], whenever he came here [CCC], we would see each other, he is the only person who knew. But at this other school, I’m still new and from the way I find them, it’s like they are rumour mongers, so I won’t say, I’ll just go then come back, get caned and the story ends (ALH FGD07)*

*…at school I haven’t trusted any teacher…that’s why I always give an excuse of a headache or stomach ache for me to go to hospital when I go (to) maintain (refill) my drugs. (ALH FGD07)*

Table 3 summarises findings on organisational policies seen as challenging and students’ reported responses to these. Of note in relation to these findings, while the Kenyan government banned corporal punishment in Kenyan schools in 2001, and enacted the Children's Act [43] which entitles children to protection from all forms of abuse and violence, corporal punishment is still used in Kenyan schools, when teachers believe it is for the child's good [44]. For ALH, maintaining silence over HIV status in this way reflects a constrained form of agency in the face of discrimination and injustice, also described in high income settings [45].

**Table 3:** Challenging organisational policies in schools and students’ coping strategies

*Challenging organisational policies*	*Coping strategies adopted by students*
• Public checking of students’ bags at the start of a new term, when medicines might be tipped on the ground • Requirements that all medicines are handed over to the matron for safe storage and dispensing • Where medicines could be stored in dormitories, locking of these during the day, and the risk of discovery during random dormitory checks • A requirement for formal permission to be out of school for health or other reasons, including attendance at Comprehensive Care Clinics, with a range of punishments if breached • A lack of teaching support and punishment for failing to ‘catch up’, on missed classes	• Storing ARVs in school toilet blocks or outdoor hiding places, requiring special effort to access privately • Putting ARVs in unmarked containers rather than labelled prescription bottles, risking confusion about identity • Accepting physical punishment for being late to class (typically, ‘caning’) to allow a student to take ARVs in private between breakfast and class, rather than explain the reason for lateness • Choosing to miss school to collect ARV refills without giving an explanation, choosing a risk of physical punishment rather than disclosure of their status; or explaining school absences as due to less stigmatizing conditions. Another approach was intentionally being the ‘naughty student’ who missed school on purpose so that no one would know when they missed school for refills • Using own time and borrowing other students’ notes to try to catch up on missed classes

The difficulties experienced by ALH in countering these challenges, for example in being seen by peers as needing regular medication, was well illustrated by one caregiver:

*They get a problem in taking the drugs at school because [other students] will see them and [they] may be asked ‘What are these drugs for?’...you see our girls they always walk together everywhere, if they want to drink water or they go to the toilet (caregivers FGD)*

School staff and policy makers also recognised the existence of stigma within schools and its consequences:

*…this somehow really jeopardises suppression because at times when this information leaks out to these other students, they tend to either make fun or discriminate… one way or the other we don’t get good outcomes. (Health policy maker 01)*

The interpersonal challenges encountered by young people living with HIV/AIDS in schools have been widely described in the literature, including in sub Saharan Africa and from elsewhere in Kenya [36–40, 42]. Our data largely support existing accounts of stigma and discrimination likely to be experienced, and here we particularly focus on the reported attitudes and actions of peers and school staff that were core to the capacity of ALH to thrive in schools. As summarised in Table 4, alongside experiences of stigma and discrimination, some staff offered remarkable levels of support, potentially offering students a lifeline. While teachers who were themselves living with HIV/AIDS might try to support affected students, they also worried about and sometimes experienced stigma and discrimination themselves. One such individual was described as being actively discriminatory towards ALH to limit risks of their own status being uncovered.

**Table 4:** Illustrative examples of interpersonal support and challenges for ALH in schools

*Discrimination/negative attitudes experienced*	*Positive support from staff*
• Teachers not allowing ALH to participate in certain activities such as games lessons and telling other students not to play with a student known to be HIV positive • Teachers discussing students HIV status amongst themselves (reported by ALH and KENEPOTE members) • Peers’ refusal to sit next to or share personal items with others thought or known to be HIV positive; broadcasting information on students’ or teachers’ HIV status, including by writing on blackboards; and ridicule • A participant who disclosed his HIV status to a close friend in confidence later entered the classroom to find his classmates discussing his status	• School staff helping ALH navigate challenges around the inspection of personal property and ARV storage, for example by undertaking ALH inspections or personally keeping; or ensuring day pupils had access to evening meals in school where food at home was known to be in short supply • Staff helping ALH to navigate challenges in schools including paying transport costs and accompanying to CCC to ensure they received refills. Some staff made sure they were in charge of bags inspection on opening days so they could avoid tipping of ARVs of students who had disclosed their status to them • One boarding school matron supported four students living with HIV from school entry for four years, by ensuring their privacy in taking ARVs, access to a good diet, including making meals in her own house, and that the girls did not undertake heavy physical work at school. Since these girls were from the matron’s home area, their ‘special treatment’ was widely accepted as a form of favouritism and did not generate stigma

The risk attached to actions taken by students to protect their privacy in social spaces was well illustrated by one female student who described her ARVs as ‘headache tablets’ to peers, and later felt obliged to share these with a friend who asked for some painkillers when she developed a headache. In the next section on experiences around visiting CCCs, we note a particular problem for ALH in accounting for the exact numbers of ARV tablets dispensed at a time, underlining additional challenges this female student was likely to face in future.

### HIV comprehensive care clinics: influences on ALH vulnerability and resilience

While the importance of well-functioning Youth Friendly Services at CCCs is clearly recognised, young people and other stakeholders in our study described a range of challenges typically encountered [46]. In contrast to the school environment, privacy concerns were less prominent since attendance implied a positive HIV status for all clients. Instead, the main forms of vulnerability described related to negative staff attitudes, often influenced by underlying resource constraints. A core challenge for young people was the time spent in clinics, due to high client-to-staff ratios. Access to specialist support, such as professional or adherence counselling, was particularly difficult since these services were available intermittently or required distant referral. During our observations, a clinician attended almost 100 clients in a day, with waiting times of up to six hours.

*P3: At times the doctor may only be one.*

*P1: Yet there are many patients, so you won’t be attended fast. You want to go back to school but there is only one doctor (ALH Group interview 01)*

Across our study, CCC services (except in the main CCC facility) were generally offered in one room where four to eight staff worked with clients across all age groups; accordingly, we noted many providers trying to communicate in ‘whispers’. When the waiting area was shared by all age groups, there were heightened privacy concerns:

*P3: When you just enter the door (of the CCC), all the eyes are on the entrance aah… you feel shy to walk up to there ….*

*P1: Others are even surprised…“Mh! A very small child using medicines [ARVs]? ...some women started talking, “Ah K’s child is also using drugs” …you know what, I don’t like it. (ALH Group interview 01)*

Given these constraints, individual CCC staff attitudes strongly influenced ALH experiences of attending these clinics and their capacity to manage lifesaving treatment. Supportive attitudes were commonly described, summarised in Table 5, including actions empowering ALH as a group as well as support for privacy, flexibility in approach towards individual young people and (often personal) contributions towards supporting ALH in difficulties:

*…adolescents are very delicate…if an adolescent says ‘I don’t want X to see me’ and then I say ‘its ok who do you want to see you?’... you know most of them think that being HIV positive is the end of their life, no so I think we just need to understand them and walk with them as they come. (Health care provider IDI20)*

**Table 5:** Individual CCC staff actions supporting ALH wellbeing

*CCC staff actions supporting ALH*
• Giving ALH a lead in planning outreach activities e.g., World AIDS Day
• Planning ALH-only CCC sessions, including during out-of-school hours/at same time as support groups to save time, transport costs and reduce privacy risks
• Waiting times at CCCs addressed by ‘fast tracking’ ALH wearing school uniforms
• Guardians/parents allowed to collect ARVs on ALH behalf for up to 2 months; ALH allowed to collect ARVs outside prior appointment times where a valid reason given; offering flexibility in clinic appointments when ALH attend on ‘wrong’ day (e.g., set appointments clash with important school events)
• Adjusting times when ALH take ARVs to fit in with other schedules eg., To coincide with school break times
• Individual CCC staff supporting ALH, including through: ‘informal’ task shifting strategies to reduce queues and allowing ALH to choose which provider to talk to at CCC
• Taking the initiative to develop and implement ART training for elderly caregivers
• Giving ALH in boarding schools enough ARTs to last to midterm or setting up a local CCC contact near to the school
• Supporting ALH privacy: visiting homes (on clinical indications) in the guise of a friend or selling items; suggesting ALH pad ARV bottles with cotton wool to prevent ‘rattling’ bringing attention to this medication
• Individual ‘emergency support’ in bringing urgent ARV supplies to homes at weekends and staff giving cash from their own pockets

At the same time, ALH and caregivers described a series of important challenges linked to negative CCC staff attitudes. A frequently reported challenge arose from a CCC policy for staff to count remaining ART pills (‘pill count’) for each attending ALH to assess ‘adherence’ to a prescribed regime. Some staff reportedly dealt harshly with young people where mismatches occurred between expected and actual counts.

*P3: The one at the pharmacy, if you go there with a lot of drugs, they will quarrel [with] you so much, I don’t know what! They will scold you… it’s like they are a teacher now, you’ll be scolded*

*P1: Yes, even if there are only two remaining.*

*P2: Even if it’s only one remaining. (ALH Group interview 01)*

Alternative explanations, such as errors in dispensing practices (which were possible explanations), seemed not to be explored. A similarly negative attitude could be associated with a finding of high viral loads, interpreted as reflecting ‘non-adherence’. Suggesting risks of staff showing negative attitude, health providers in one CCC reportedly chose to use a back door to enter the facility to avoid public recognition, which was tellingly referred to as the ‘stigma door’. As suggested earlier, resource constraints underpinned many structural challenges for CCCs, particularly staff levels and skills and physical space. In this way, when some trained adherence counsellors were described as ‘reprimanding and blaming’ rather than supportive, these behaviours could also be seen as signs of ‘burnout’ linked to high workloads:

*… if a youth comes today and it’s not his/her clinic day and the way I am already tired, your head is not working well, you can respond that youth badly. When you respond rudely to the client, you risk losing him/her. (Health care provider IDI 03)*

### AHOS research participation: influences on ALH vulnerability and resilience

To inform a deeper discussion of the ways in which research participation might potentially counter and exacerbate the everyday vulnerabilities described for ALH so far, in this section we describe findings around ALHs’ experiences of being in AHOS, focusing on two core emerging themes around decision-making on joining the study and experiences of study procedures.

#### Deciding to join AHOS

The approach to informed consent and assent for AHOS (see Additional file 1) aimed to take account of the complex social, cultural and legal influences on ALHs’ actual and assumed capacities for independent decision-making, reflecting agency. Influences included their emerging autonomy and age, given cultural and legal variations in assessments of maturity and around human rights [9, 47]. Additional recognised challenges for AHOS included risks of inadvertent disclosure in approaching a young person who might be unaware of their HIV status and the need to seek consent from both caregiver and their teenagers as independent participants.

In practice, a research team member approached adults living with HIV who were known to be parents of ALH during routine CCC visits to explain the study, establish their initial interest in being involved and ensure that only ALH who were aware of their HIV status were invited. With initial parental support, an AHOS team member approached ALH eligible to join the study either at CCC or at home, to explain and discuss the aims and activities involved in participation and assess interest. To build trust in the study, including around privacy, this team member had been recruited as a person known to CCC-users through an earlier role as a volunteer at a CCC and with an NGO working with families and adolescents to support ART adherence. The formal AHOS consent process, including assent from the young person and consent from their caregiver or guardian, took place in separate spaces at the AHOS research centre prior to the first study assessment, with confirmation at subsequent annual visits.

Throughout our study, we noted the positive relationship between AHOS participants and this—and other—AHOS team members, including young people’s willingness to listen to their advice. The formal AHOS consent process, including assent of the young person and consent of their caregiver or guardian, took place immediately prior to the first research clinic-based assessment, when the young person and their caregiver travelled to the research clinic.

While there was reasonable clarity amongst ALH about the broad aims of and study procedures in AHOS, some young peoples’ and caregivers’ existing fears, such as worries about their ‘thinking abilities’ linked to memory and concentration, positively influenced some to join, as a way of accessing more information or advice. In some cases, a combination of parental authority traditional in this community and an assumption that participation might be beneficial seemed to generate prompt decision-making to join [47]:

*[How did you learn about the study?] I was told by my father over the phone … he told me to come the following day, I was needed at KEMRI Kilifi, and I went because I know my status. (ALH FGD02)*

#### Experiences of AHOS procedures

Over a two-year period, participating families attended three annual research visits, each lasting half a day. As described in Additional file 1, AHOS research procedures involved anthropometrics, clinical examination, blood sampling and responding to a series of questionnaires, using audio-computer assisted self-interviewing (ACASI) and a face-to-face interview.

Perhaps the most common and strongest accounts around experiences of AHOS participation concerned the value young people placed on the open and friendly attitudes encountered at the research clinic, including reassurances around privacy:

*They [staff] keep secrets…at the beginning you will be told there is no one who will know whatever you have said and it’s true no one knows. It’s only you and the computer…and the doctor…you feel very good (ALH FGD02)*

*Whenever I go there, I feel happy because they treat me nicely…when you go there everyone is free with you…there is a way you can ask someone something and their reply is cold that…I will feel afraid and even think of not coming back…but since they [KEMRI] treat me nicely, even as I say “hello” to their phone calls, I immediately dress up and go (ALH FGD02).*

One ALH described the importance of feeling ‘noticed and remembered’ through setting up a study like this, focused on the needs of ALH. Also of great value to young people was the opportunity offered to meet peers, some of whom might be dealing with similar life challenges, with opportunities to share coping strategies.

In general, ALH saw physical examinations as helpful, being more in-depth than those encountered at CCC, and the clinical staff undertaking examinations as respectful and engaging, rather than patronising or harsh. While some ALH found aspects of interviews, tasks and questionnaires ‘childish’, irrelevant or embarrassing, these procedures were generally seen in a positive light, as being individually informative and generally enjoyable. Since many ALH did not have regular access to computers at home or school, the ACASI-based tasks were often seen as an exciting “computer game”. Young people also viewed research activities, particularly cognitive assessment tasks, as a form of learning, with a potential to compensate for missed classes:

*I really thought about it, I miss school to come here?... When I got here, I found the questions are the same as those in school so I felt good. Now I wonder if I had not gone, would I really get them [understand questions in school] or I would miss them? (ALH Group interview 01)*

At the same time, challenges emerged for ALH related to the time taken for AHOS appointments and difficulties experienced in explaining their participation to others. In relation to time, although research visits were much less frequent than routine CCC appointments, getting time out of school to attend AHOS appointments generated similar risks and challenges, including anxiety, risks of punishment linked to actions taken to avoid inadvertent disclosure and additional workloads in catching up missed schoolwork.

*P2: If it’s on a school day and they [KEMRI] have called me, I will go the following day… I won’t go to school the following day, I already know that. I will come up with a lie because once you go there (to school) leaving is a no, he/she cannot allow you to leave. (ALH FGD03)*

*P6: If they [KEMRI] call today, I won’t go to school the following day. I will just go…and know how to approach that teacher. If he/she canes me I will persevere [with] the pain and move on. (ALH FGD03)*

Importantly, following an AHOS appointment, young people experienced challenges in explaining the study (and their school absence) to others without risking disclosure of their HIV status. The study information sheet used in schools avoided reference to HIV to counter such privacy risks, instead talking about research on ‘cognitive functioning’. In practice, class teachers often asked for more information about AHOS that ALH were ill placed to give without risking disclosure. The term ‘cognitive functioning’ also generated teasing as meaning ‘mental health problems’, which already vulnerable ALH found difficult to manage. As a result, in some instances ALH left school without permission to attend AHOS appointments, risking punishment for being absent without a good reason. As for CCC visits, they would also need to catch up on missed lessons in their own time.

## Discussion

### Risks of cascading vulnerability in everyday life

Across earlier sections, we have shown that sources of vulnerability and resilience for ALH in our study were interrelated and cross sectoral. In this way, access to social and economic resources importantly underpinned diverse, individual experiences of vulnerability and capacity for resilience. Thus, for example, micro-level policies in schools around ART storage and access and permission to attend CCCs prompted actions to protect privacy that could have perverse impacts on wellbeing. Maintaining silence over HIV status emerged as a response by young people to acts of discrimination and injustice, reflecting constrained forms of agency [29]. Missing ART doses, taking time out of school without permission and accepting unfair punishments were detrimental to physical, emotional health and educational progress, which in turn could increase privacy risks and progressively worsen social, health and educational outcomes. Organisational policies at CCCs, intended to promote ART adherence, but potentially enacted in harsh ways, similarly undermined emotional wellbeing and could chip away at motivation to attend CCC or take ARTs regularly [46]. Long waiting times at CCCs exacerbate the challenges in being away from school (for school-going ALH), as would the lack of underlying family support. Efforts and investments are clearly needed across different policy sectors to improve ALH’s health and well-being, but inter-sectoral linkages are reportedly challenging to establish, undermining efforts to promote ALH welfare.

Our findings also illustrate young people’s resilience through less constrained but still potentially harmful forms of agency adopted by ALH in response to anticipated or enacted stigma. In this way, and as highlighted in the literature, ALH agency may be constrained or bounded by structural constraints within their context [18]. Examples in our data include accepting less stigmatising health labels (such as epilepsy), sharing ARTs to support a claim to friends that these were headache tablets, switching CCCs (or schools) and refusing to be seen by certain CCC staff. We also see forms of what might be described as more positive agency, particularly through the actions of adherence champions’ at CCCs, that is, young people who are living with HIV who choose to be open about their status to support other ALH. Notably, the actions of adherence champions reflect forms of relational agency wherein young people’s healthy choices depend importantly on the support of key others, often involving a highly focused and supportive strategy, initiated and maintained through NGO support to NASCOP offices.

While these experiences of vulnerability reflect enormous challenges for affected young people who are trying to make their way in the world, we heard many positive accounts of youth-friendly policies and individuals working to promote the resilience and wellbeing of young people living with HIV in school and at CCCs. In relation to AHOS, we were struck by young people’s delight in and appreciation of the friendly and respectful manner encountered in the research team and opportunities to spend time with their peers. The experiences of participating in AHOS stand out as instances of being respected, listened to and supported, made more important by the fears and experiences of stigma and blame encountered in many areas of their lives, particularly where underlying conditions of poverty and insecure family structures and relations are faced at home. In this sense, ongoing participation in a carefully designed and supportive study offered a kind of respite from daily life, while offering tools and a source of support to better cope with challenges in daily living. Overall, research participation may have increased some forms of vulnerability for ALH—particularly those related to taking time out of school or being unable to answer questions about AHOS—but it seems likely to have promoted resilience for many young people. The value of study participation also raises difficult questions about what happens once a study ends.

Notably, the retention rate for AHOS has remained above 95% across the first year of its duration, supporting an interpretation that participation is valued. In studies where recruitment and retention rates are high, questions might be raised about the appropriateness of benefits involved. We argue that AHOS provides an example of a situation in which high retention rates and appreciation of resources offered do not imply that the benefits provided are ‘too much’. Rather, the implication is that the everyday lives of this group of young people can be so challenging that their interactions with and support from researchers, which might be regarded as normal outside a context of such marked vulnerability, are instead interpreted as importantly self-affirming.

#### Strengths and limitations of the study

In presenting this discussion of study findings, we have been cognisant of the need to maximise trustworthiness of our approach, including taking account of potential influences across the research process [25]. For example, our choice to include HIV champions and mentor mothers in our early data collection activities around ALH experiences meant that we did not hear the voices of the most vulnerable young people directly, instead drawing on the accounts of others involved in their support. A wider group of ALH were involved in the later workshop, but these discussions took place in groups, which may have impacted openness. Mentor mothers, community-based research staff, health managers and policy makers involved in the study were able to draw on their longstanding and in-depth experiences of working with families impacted by HIV/AIDS in this setting to support our understanding of potential vulnerabilities and forms of resilience.

Across all phases of the study, we took care to ensure that members of the research team likely to have greatest rapport with different participants (for example, through age, culture and language) collected data from these individuals and groups. The research team involved social scientists and community engagement specialists, and a majority (except one individual) were long-term residents in Kilifi. Team debriefing discussions followed all data collection activities. Given the focus on informing policy, we used a Framework Analysis approach to support transparency in the data analysis and interpretation, including through regular team discussions.

### How should researchers’ respond?

A central ethical obligation within research practice is to minimize risks to participants, in reasonable balance with the study’s potential benefits. When participants are especially vulnerable at baseline, in their everyday lives, greater care must be taken not to make matters worse through research but, ideally, improve their situation. Our analysis reveals a challenging situation in determining how best to respond to the adolescent’s needs in the research context, where researchers and staff become intimately aware of life risks and vulnerabilities beyond the scope of the study. The analysis illustrates how multiple influences within and across sectors can interact as ‘layers’ of vulnerability that generate steadily worsening forms of vulnerability for ALH, leading to clusters of increasingly serious physical and mental health, social, educational and economic outcomes. At the same time, in our data, vulnerability cascades were potentially reversible, so that positive inputs within and across sectors have the capacity to build increasing resilience and positive outcomes across these clusters. This raises a central ethical question about the appropriate scope of researchers’ responsibilities towards potentially vulnerable individuals they hope to involve in studies: how should studies be designed to be responsive to unique and overlapping risks, given that carefully planned research is key to identifying evidence-based interventions and policies that might intervene on reversible risks of vulnerability?

In making arguments for researchers’ responsibilities to understand and address specific risks of vulnerability and vulnerability cascades, it is first important to note some limitations for our study that may underestimate the underlying risks and vulnerabilities of ALH. Firstly, the most in-depth accounts of ALH vulnerability are not derived from young people themselves but other stakeholders, as a deliberate strategy to maximise the extent to which their involvement was likely to be a positive or empowering experience and avoid increasing burdens for the most vulnerable in this group. Further, young people in this study were in school, while many affected teenagers may not have this opportunity, given health and socioeconomic challenges. Taken together these points suggest our analysis may be based on an underestimation of the burdens generally experienced by ALH in our context.

With that in mind, Luna’s (2019) analysis frames researchers’ responsibilities towards potentially vulnerable participants as a series of steps to characterise potential vulnerabilities in relation to their nature, their seriousness and probability, and assess researchers’ responsibilities accordingly. The most serious forms of vulnerability, including cascading forms, should be most urgently addressed, acknowledging that these can sometimes be the most difficult to address when involving deeper social and political drivers [17]. Further, we have shown that sources of vulnerability and resilience may only become clear once a study is in progress, highlighting an important gap for global health research ethics in the ‘post ethics approval’ space, and the importance of embedding reflective ethics support and research to inform responses [48]. Critically, in responding, researchers should also seek to empower and strengthen the resilience of otherwise vulnerable participants as much as possible, to balance against concerns of paternalism and ‘victimisation’. Drawing on the literature and the example of AHOS, in the following paragraphs, we discuss two broad responses that seem important for researchers working with ALH in this and similar settings. Following these sections, in Table 6, we propose a set of recommendations for researchers planning studies involving ALH, particularly applicable to resource-constrained settings.

**Table 6:** A summary of emerging recommendations for research involving ALH

**Overall**	
Researchers have an ethical and professional responsibility to plan studies involving ALH through systematic processes of ongoing engagement with key stakeholders to optimise research, feed into cross-sectoral government and non-government policy development, and to seek opportunities to support young people’s resilience in the short and longer term. Embedding ethical reflection and empirical ethics research within studies can strengthen the rigour and effectiveness of stakeholder engagement, through contributing a greater depth of understanding of forms of vulnerability and resilience for ALH in a given context, and how these may interact with proposed research approaches. Sensitively designed research may also help inform health and social interventions that disrupt patterns of vulnerability for ALH	
*Key finding*	*What should researchers do?*
**Community/home**	
Community-wide **stigma/discrimination** is reportedly lessening over time but remains widespread, including that adolescents living with perinatal HIV/AIDS infection experience stigma through assumptions of ‘immoral behaviour’ as a result of vertical transmission of HIV/AIDS being less widely understood	• Support government and NGO in planning public engagement on HIV/AIDS and ALH, including vertical modes of transmission, while taking care not to increase stigma around ALH with horizontal transmission • Support involvement of ALH, their families and other key stakeholder in planning public engagement strategies • Work with individual ALH to develop plans for engaging family members, recognising there may be stigma within families
**Poverty** is a core structural cause of harm for ALH, including through impacting access to education, a healthy diet, health care at CCC, accurate timing of ART doses and, for girls, ways of managing menstruation, which in turn impacts schooling. Education is further impacted where ALH leave school early to seek paid employment or are forced to wake very early to walk to school **The role of the family** is core to the risk of ALH experiencing vulnerability and/or resilience, for example through providing moral and practical support to attend CCC appointments, take ARTs on time, and accept and live positively with their status. In practice, family structures and attitudes to HIV/AIDS are diverse, with over half of ALH in AHOS being orphans living with guardians, so that inadequate family support is an import risk for increasing cycles of vulnerability	• Establish effective ancillary care pathways through links with government services, NGOs^a^ supporting poverty alleviation (food distribution, etc.) and through working with young people and their families to support decision-making around financial and economic challenges faced • Ensure participation is practically supportive or, at the very least, cost neutral and look for ways to provide additional support to ALH, such as providing nutritious hot food during clinic visits; ensuring reimbursement of travel accounts for costs and other burdens, e.g., takes account of meals missed while travelling • Link with peer networks, local NGOs and government social services to help connect ALH and their families, including ALH living outside families, with additional social support. (Note below that ALH in boarding schools may also benefit from additional social support.)
**School**	
A core concern for ALH in school who join studies is increasing risks to their privacy, and stigmatisation by staff and students, leading to actions that increase vulnerability through impacts on physical and mental health and educational progress (as a ‘cascade’)(see Tables 3 & 4). Important examples include that: • Typical systems of storage and gaining access to medicines in schools may challenge ALH privacy and timely use • Being out of school to attend CCC and/or research clinic appointments increases existing privacy risks for ALH who need to explain and seek permission for their absence (see Table 3). Strategies adopted may generate harm in the short and longer term, including physical punishment and loss of educational opportunities • Boarding schools may be particularly challenging where ALH lose regular access to positive family support	• Undertake and draw on findings from stakeholder engagement and empirical ethics research to build ALH resilience and reduce risks of increasing vulnerabilities. Examples include: ○ involving ALH and KENEPOTE teachers in designing study information sheets for schools/head & class teachers; ○ supporting ALH in studies to be able to explain to peers and teachers what the study is about without undermining privacy or increasing stigma; ○ ensure support is available to ALH joining studies to catch up on missed lessons, without increasing risks of stigma; ○ designing studies to minimise ALH time out of school Ensure ongoing dialogue and end of study feedback of findings with stakeholders in the education sector (policy and school based) to influence policy and practice. Examples include supporting teaching staff to better understand ALH dilemmas (see Tables 3 and 4) and seek ways to strengthen relations with, and between, students more widely, to address stigma at these levels
**HIV/AIDS Comprehensive Care Clinics**	
Typical and interrelated concerns for ALH attending CCC were linked to structural resource constraints and staff attitudes, and less to privacy risks	• Ensure ongoing dialogue and end of study feedback of findings with stakeholders in the health sector (policy and CCC based) to influence policy and practice, including: ○ Highlighting issues around ALH privacy and staff attitudes that relate to resource constraints, including ways that staff work positively around these constraints ○ Promoting the wider adoption of Youth Friendly HIV/AIDS clinics to minimise time spent out of school for school-going ALH and promote ALH resilience more widely ○ Giving feedback on perverse outcomes of policies in practice, such as ‘pill counting’ ○ Identifying the role that communication and communication skills training can play in supporting health workers and their clients
**Research studies**	
Research participation offers an opportunity to support ALH emotionally and practically through health checks, supportive staff attitudes, providing nutritious meals and meeting up with peers in a safe and friendly space Research participation presents a vulnerability risk to ALH in schools through the time lost from school when attending research appointments. It is important for researchers to recognise that ALH routinely lose time out of school for health reasons, including ill health and attending CCC, in addition to time spent in research participation Research participation may increase existing vulnerabilities through risks to ALH privacy through study information leaflets or being asked to talk to teachers or fellow students about their participation Researchers’ responsibilities to protect participants’ privacy, and ensure participants feel confident about these measures, is likely to be supported where they are approached and recruited by a trusted individual. At the same time, a relationship of trust may influence decision making around joining the study in ways that could undermine full autonomy	• Researchers should recognise the important role they can play individually in building ALH confidence through positive interpersonal interactions during the research encounter. Study planning and staff training should aim to maximise this opportunity, without creating emotional dependency • Participation in research can increase privacy and stigmatisation risks, especially for ALH in school, through researchers’ and students’ communication about the topic of the research (HIV/AIDS) with teachers and other pupils before, during and after the study. Researchers should design and pre-test all written materials (such as information leaflets) carefully to avoid this risk. The research team should also support ALH to be able to talk about the study and their participation in ways that do not imply their HIV status or other health problems. ALH should be involved in processes of developing and pretesting materials and other approaches to limiting risks of stigmatisation • Study planning should center on minimising time that ALH spend out of school to attend research clinic appointments, seeking to limit the time needed and the frequency of appointments as well as planning activities to coincide with school holidays as much as possible • Researchers should recognise, weigh and seek ways to address the risk that trusted individuals who are involved in leading informed consent processes may inappropriately influence potential participants’ decision-making. Appropriate steps include making sure that the individual responsible for seeking informed consent is aware of this risk, is trained to minimise the risk, and that there is no pressure placed on them in relation to numbers recruited over time • Informed assent/consent processes for ALH who are minors, and their parents/guardians, should take careful account of existing family dynamics and seek to ensure that the young person is independently informed and willing to participate. Informed consent should be a process in which researchers can assess understanding and willingness of the young person to join

^a^Support from NGOs and government are in place, but former have fixed funding cycles and latter have many competing funding requirements

#### Grounding privacy strategies for research in ‘real life’ experience

Given the risks of cascading vulnerability in everyday life, our data underscore that potentially the most important emerging ethical concern for research involving school-going ALH is ensuring that study participation does not increase existing privacy risks, including the need to provide or discuss reasons to be out of school that implicate their health. Added to the community engagement processes used to support AHOS, an important practical step could include greater engagement of ALH in developing strategies to support young people’s capacity to talk about study participation in ways that do not risk a privacy breach in everyday life. While we involved Kenya Network for Positive Teachers (KENEPOTE) members in our learning about the experiences of ALH in schools, we recognise that members of this group could also have helpfully contributed to study planning.

While protection of young people’s privacy was core to ethical practice in AHOS, we note that strategies developed to protect young people’s privacy during the informed consent process could generate an ethical challenge. ALH and their families already knew the individual undertaking much of the engagement for the study, as an HIV/AIDS community support volunteer. As well as providing reassurance to families around privacy concerns, this strategy seems likely to have built trust in the research process itself, potentially offering some risk to young people’s autonomy given the close relationship of trust already in place. Luna’s (2019) emphasis on prioritising the most serious and urgent challenges for ALH suggests that a good balance was made in focusing on critically important privacy risks for ALH and their families, particularly given its evolving characteristic in this age group [9]. This dilemma illustrates the critical importance of ensuring that individual(s) in the team undertaking consent and interacting closely with ALH and their families have a high awareness of these autonomy risks, and – critically – the skills and institutional support to manage these.

#### Responding to structural injustices

Overall, our findings show very clearly that the most serious forms of cascading vulnerability faced by ALH in AHOS are largely structural and unrelated to research participation. Instead, existing forms and acts of stigma and discrimination, and the strategies young people adopt to contain these risks, combine across different domains of life to generate vicious – or potentially, virtuous – cycles. While AHOS participation was generally seen as a positive experience, time spent on study participation – including travel – may clearly contribute to vulnerability, when added to the challenges experienced in attending CCC. Importantly, an unpublished interim analysis within AHOS has identified significantly poorer outcomes in cognitive, educational, and mental health across quantitative assessments for perinatally infected ALH in comparison to control groups. While educational outcomes are likely to reflect cumulative biological, social and economic influences in affected young people, it seems particularly important to limit time lost from class through CCC attendance and research participation. Ideally, research visits should take place at weekends, or during school holidays, but this strategy may not be practical in time-limited large cohort studies. There may be no ideal solution to this dilemma, but researchers should actively seek ways to minimise time taken out of school as well as limit risks of unintended adverse outcomes, including threats to ALH privacy and inappropriate punishments or sanctions. Additionally, the risks and burdens of school-going ALH spending time at research clinics, even if infrequent, must be assessed alongside other reasons for school absence, as a cumulative phenomenon with important implications for their wellbeing. As summarised in Table 6, the main implication for research concerns the need for wide engagement as part of study planning and prioritisation of participant interests.

In the short-term and more narrowly, researchers are argued to have ‘ancillary care responsibilities’ in relation to a range of reasonable forms of unmet health and social care need that participants experience during studies, including through setting up public sector and other referral pathways in advance of studies [49–51]. Within AHOS, many short-term health care needs for ALH are addressed directly during clinical research assessments (such as prescribing courses of antibiotics) and there are well-developed referral systems to public sector partners for other health and social care needs (see Additional file 1). At the same time, public sector referrals do not cover all needs, sometimes leaving young people and their families unsupported or research teams to support in ad hoc ways that may generate ethical dilemmas for front line staff, for example, giving personal support that may unduly influence ALH decisions to participate and may be unsustainable. We argue here and elsewhere that researchers’ ancillary care responsibilities in low resource settings should be based on a careful understanding of local context, including diverse stakeholder views, and developed as part of a structured, accountable institutional response to the needs of study participants and front line staff [48].

In addition, our study reinforces a well-recognised responsibility for researchers to contribute to tackling underlying structural causes of unmet needs impacting the communities where they work [49–54]. The preliminary findings from AHOS on poorer outcomes for this group of young people clearly illustrate how thoughtfully designed research can help contribute to critical knowledge around the layered vulnerabilities of a population, like these ALH in Kenya. In this way, by helping to inform interventions targeting biological and social sources of vulnerability, sensitively designed research may help intervene or interrupt vulnerability cascades. The nature and extent of researchers’ responsibilities in relation to existing forms of structural inequity may depend on the context, including the nature of the research and its social value, and the opportunities presented by the history and depth of researchers’ involvement in research in a given setting.

Specific to AHOS at KWTRP, a series of mechanisms aim to support the social value of research, including government and non-government stakeholder consultative workshops before and throughout the study, one element of which was a Young Persons Advisory Group drawn from local secondary schools in Kilifi [55]. In addition, scientific governance mechanisms at institutional and national levels assess the social value of ethically challenging observational studies (such as AHOS), such as opportunities to move rapidly towards policy or future intervention research that can more directly inform policy and support social value [47, 56–58]. The continuing task for all researchers is to identify meaningful ways of supporting potentially vulnerable individuals and populations in ways that support their fair involvement in studies, recognising the potential of careful empirical research as well as wide and meaningful consultative activities to inform such measures. While these relationships may be easier for well-established research institutions to build, a challenge emerges that the most vulnerable populations are likely to reside in remote areas without the infrastructure needed for collaborative research.

## Conclusion

The potential for experiences of research to exacerbate vulnerability and strengthen resilience in the existing lives of young people living with HIV/AIDS, including through positive or negative cascades, has important implications for research planning. Drawing on Luna’s 2019 account of vulnerability in research ethics, this empirical ethics study in coastal Kenya details the complex ways in which contextual, organisational and interpersonal influences at home, in the community, at school, in health care and in biomedical research settings may interact to impact the lives of young people living with HIV/AIDS who join studies. The findings importantly contribute to critical knowledge around the layered vulnerabilities of a specific population, like these ALH in Kenya, helping to inform targeted interventions across the disciplines of health care, education and social care as well as health research. Strong and on-going relationships between researchers and policy makers are critical to promoting the uptake of empirical ethics research findings into policy and practice. We argue that researchers’ responsibilities include addressing structural causes of vulnerability for ALH research participants, giving examples of strategies, and for the importance of embedded empirical ethics research to identify context-specific risks and opportunities.

### Supplementary Information

**Additional file 1.** A summary of the Adolescent Health Outcomes Study (AHOS).Provides detailed information on components of the Adolescent Health Outcomes Study in which this empirical ethics research was embedded, including procedures for engagement and informed consent, research activities and ancillary care plans.

**Additional file 2.** Data collection tools used across the study. Includes the tools used for data collection across the study.

## Data Availability

The datasets generated and/or analysed during the current study are not publicly available due the sensitivity of the data and the potential that aspects of this data set may infer identity of individual participants and may reflect sensitive areas in individuals’ lives. Data will be made available from the corresponding author on reasonable request.

## Abbreviations

ACASI: Audio computer-assisted self-interviewing
AIDS: Acquired Immunodeficiency Syndrome
ALH: Adolescent Living with HIV/AIDs
AHOS: Adolescent Health Outcomes Study
ART: Antiretroviral Treatment
CCC: Comprehensive Care Clinic (for HIV/AIDS)
FGD: Focus group discussion
HIV/AIDS: Human Immunodeficiency Virus/Acquired Immunodeficiency Syndrome
IDI: In-depth interview
KEMRI: Kenya Medical Research Institute
KEMRI SERU: Kenya Medical Research Institute Science and Ethics Review Unit
KEMRI CGRMC: Kenya Medical Research Institute Centre for Geographic Medicine, Coast
KENEPOTE: Kenya Network for HIV/AIDS Positive Teachers
KWTRP: Kenya Medical Research Institute-Wellcome Trust Research Programme
NASCOP: National AIDS and Sexually Transmitted Infection (STI) Control Programme
NGO: Non-Governmental Organisation
OxTREC: Oxford Tropical Research Ethics Committee
YFS: Youth Friendly Services

## Footnotes

1 From a preliminary unpublished analysis of AHOS data, 52% caregivers described their ward as an orphan.
