# Supplementary material for: Layered vulnerability and researchers’ responsibilities: learning from research involving Kenyan adolescents living with perinatal HIV infection
Source: BMC Med Ethics. 2024 Feb 20;25:21. doi: 10.1186/s12910-023-00972-3 (PMC10877892; doi:10.1186/s12910-023-00972-3)
Supplement: Supplementary file 1 — Additional file 1. A summary of the Adolescent Health Outcomes Study (AHOS).Provides detailed information on components of the Adolescent Health Outcomes Study in which this empirical ethics research was embedded, including procedures for engagement and informed consent, research activities and ancillary care plans. [file 12910_2023_972_MOESM1_ESM.pdf]

AF1: Summary of the Adolescent Health Outcomes Study (AHOS)

The Adolescent Health Outcomes study (AHOS) is a longitudinal observational cohort study aiming to assess the impact of perinatal HIV infection on cognitive, educational and social outcomes for ALH in Kilifi county. The study involves 550 adolescents between 12-17 years and their primary caregivers, including 201 ALH with perinatal HIV infection, 157 who have been perinatally exposed but not infected with HIV and a control group of 200 adolescents with neither infection nor exposure to HIV.

As part of planning for AHOS, the Neurodevelopment Research Group drew on experience of working on child and adolescent health - including HIV - for many years in this setting, in partnership with the Kilifi County Health Offices, and County Education Offices in Kilifi. Core ethical issues recognised *a priori* were the need to:

- Ascertain that ALH were aware of their HIV status before inviting participation
- Maintain privacy for ALH and their families and provide reassurance about these protections throughout the study
- Minimise time spent by ALH in participating, particularly time away from school
- Ensure that any burdens involved in participation were limited as far as possible and adequately compensated (see Box 2)
- Plan ahead for ancillary care (health and social care) needs that might arise during the study.

Engagement and informed consent processes

- Both ALH and their main caregiver were independent participants in AHOS, as noted below. Generally, young people were invited to participate during routine CCC visits, where their perinatal HIV status was already known, and their main caregiver approached at CCC or at home subsequently. Alternatively, the main caregiver was approached first at CCC and the young person later, again, either at CCC or at home.
- One of two AHOS staff leading on recruitment had main responsibility for home visits, and much ongoing interaction with families. This staff member had previously worked at the main CCC in Kilifi County Hospital and was well known to those involved, including as living with HIV, having offered psychosocial support at CCC and at home for over 10 years.

- AHOS information sheets and consent forms, and engagement with schools in general, did not explicitly mention HIV, but focused on health issues for adolescents (leading to its title of 'Adolescent Health Outcomes Study').
- Young people and their caregivers gave formal written consent and assent to participate at the first visit to the Neuro-Assessment (NA) Centre at KWTRP, where the main study activities were undertaken.

Research activities for ALH

The research neuro-assessment clinic is situated in the grounds of KWTRP and Kilifi County Hospital, close to the main HIV Comprehensive Care Clinic in the county. Over a duration of 24 months' participation, ALH attended the research clinic three times at annual intervals, with direct and indirect costs of participation covered and food and drinks provided during the research visit, which often last more than half a day including travel. At each visit, research procedures for young people involved a series of neurocognitive assessments, computer assisted interviews on health risk behaviours, clinical assessments of physical and mental health, blood sampling and individual interviews.

Research activities for ALH caregivers

During each study visit, ALH caregivers were invited to participate in structured interviews focused on physical, psychological, social and educational aspects of their child's development over time. They also answered questions on their own mental health and quality of life.

AHOS ancillary care plans with prior national science and ethics approval

Where a young person was found to be in need of specialist help or intervention, the AHOS team provided feedback to the parent regarding the identified area of concern and referred the young person to the relevant professional body. Referral systems between this longstanding neurodevelopmental research team and health specialists in the public sector and within Community Based Organisations had been developed over many years of interactions, including:

- Education and Assessment Resources Services (for example, for children with severe learning problems)

- Ear, Nose and Throat specialist clinic (for example, for hearing problems)
- Ophthalmology specialist clinic (for visual problems)
- Visual problems –Eye unit
- Physiotherapy / Occupational therapy at Kilifi County Hospital or referral to the Association for Physically Disabled in Kenya (for developmental delay and motor problems)
- Community Based Organisations (for family/financial e.g. supports the education of children with disabilities)
- Neurology specialist clinic (for neurological problems)
- Psychiatric unit or social welfare department (for mental health problems)
