# Supplementary material for: Layered vulnerability and researchers’ responsibilities: learning from research involving Kenyan adolescents living with perinatal HIV infection
Source: BMC Med Ethics. 2024 Feb 20;25:21. doi: 10.1186/s12910-023-00972-3 (PMC10877892; doi:10.1186/s12910-023-00972-3)
Supplement: Supplementary file 2 — Additional file 2. Data collection tools used across the study. Includes the tools used for data collection across the study. [file 12910_2023_972_MOESM2_ESM.pdf]

1. **TOOL FOR IN DEPTH INTERVIEWS WITH HEALTH POLICY/CARE PROVIDERS**

Introduction

- *Thank you for agreeing to talk to us today. We are working on a study that's looking at different aspects of the lives and experiences of adolescents in Kilifi, between the ages of around 13 to 18 years. Our study aims to feed into planning of future research that involves adolescents, to make sure that research plans take account of this group's particular perspectives. In particular, we want to look for ways to ensure that research in future does not cause any 'hidden' problems, and does provide support to adolescents in areas where it can. Overall we would like research plans to be more 'adolescent friendly'.*
- *One particular area of interest in our study is understanding the everyday lives and experiences of adolescents living with HIV/AIDS (ALH), including those born with HIV and those who became infected later [refer to PMTCT if relevant].*
- *We realise that you have a lot of experience in supporting families living with HIV through providing services for diagnosis, treatment, monitoring and counselling, so really appreciate learning more about this from you. We understand that families' situations are very diverse, and that some will experience more challenges related to living with HIV/AIDS than others. We would like to learn about the range of experiences common in Kilifi, including for those living with HIV that find positive strategies to manage their situation, and others that find this more difficult.*
- *During the interview, when we talk about 'issues' faced, we would like to understand what impact these issues have on people's lives, including over time. We would also like learn how adolescents and/or families tackle issues, and how well the approaches they use work. We understand that sometimes the way people tackle challenges might turn out to be very helpful but sometimes may not really help at all.*

a) Could you tell me about your own history of working in the area of HIV/AIDS with youth?

- When did you start, where have you worked and what made you decide to work in this area?
- What are the major changes that you've seen over this time, including in services available? (*Seen as positive or negative, any why?*)

b) Policies & guidelines in the health sector

We would like to understand the different policies in health, social care and education that might affect ALH in Kilifi. We are aware of the Kilifi County HIV & AIDS Strategic Plan 2016-2020 and the NASCOP Guidelines for treatment & prevention 2016.

- Could you tell us about any other health policies you think are relevant? Can you explain what these guidelines cover – what are the main areas of recommendations?
- Can you say something about the extent to which these guideline/documents (including NASCOP and Kilifi County docs above) have been useful, in your experience?
  - Which areas are useful, and why?
  - Which areas are not so useful, and why not? [*Probe for challenges in implementation*]
  - Is there anything important missing?

c) **Health related services**

For people living with HIV in Kilifi County: What governmental and non-governmental health-related services are available that are focused on ALH particularly? [*Any documentation we could look at?*]

- Which stakeholders are involved in supporting these services (e.g. MoH, Afya Pwani, Plan etc) and how (exactly) do they contribute? [*Draw links between govt and NGOs involved – how coordinated*]
- How well are these services working – what **successes** and **challenges**/gaps exist for i) provision and ii) uptake? *Can you give examples?*
  - o *How common are these issues – can you identify areas you think are important and common?*
  - o *What is the impact of these challenges on ALH and their families; and how do they tend to respond?*
  - o *Can you talk about the way that these kinds of responses might affect quality of life for ALH & their families – both positively and negatively?*

- *[if not raised] Probe for youth-friendly services* – to what extent do these exist AND seen as important/why? Any challenges in establishing?
- In your opinion, what are the most important ways that health services for ALH should be supported or strengthened/expanded, and why? What resources would this need?

d) **Challenges for families living with HIV** *[Remind participant about main purpose of interview – review intro]*

We have talked about the support provided and challenges faced by ALH in accessing health services. Can you now tell me something about the families/ALH that you know about/are attending this clinic at the moment? What are the main kinds of challenges they face, including <u>at home/in the family</u>, <u>at school or in the community</u>? I would like to hear about these issues and the ways people handle them that you think are positive or negative.

<table>
<tr><th>Possible influences</th><th>Probes</th></tr>
<tr><td colspan="2">IN THE HOME</td></tr>
<tr><td>
• Access to basic needs for family (shelter, food/nutrition, clear water, hygiene, menstruation for girls, economic status & changes in economic status e.g. from losing jobs or costs of accessing care)
• Nature and quality of relationships within the family (supportive/unsupportive) – parents, grandparents, siblings, extended family (positive and negative relationships: e.g. Loss of parents/abusive relationships/change of parents OR positive examples of living with HIV?
(May come up here or elsewhere…)
• Health/physical wellbeing (adolescent and others in family e.g. infection/stunting)
• Psychological wellbeing (adolescent and others in family e.g. depression, anxiety, loss of concentration?) - (probe for ‘acting out’ = high risk behaviour in relation to sexual activity, alcohol & drug use, other risks; and lack of progress at school)
• ‘Personality’ [Any external events affected these situations? E.g. Human rights issues, political instability, natural disasters, strikes, disease outbreaks]
</td><td rowspan="3">Which challenges/issues are common/ important?

What are their impacts, and how does this differ between families?

What steps do ALH/families take to manage these issues?</td></tr>
<tr><td>OUTSIDE THE HOME: SCHOOL/OTHER SOCIAL GROUPS</td></tr>
<tr><td>
• Nature and quality of relationships outside home (supportive/unsupportive including STIGMA):
    o in school - friends and teachers/school nurses etc
</td></tr>
</table>

○ outside school – other peer groups e.g. church youth groups, sports clubs etc<br>○ boyfriend/girlfriend relationships<br>○ social media<br>• At school/access to learning: attendance, concentration, progress and inclusion at school<br>*(May come up here or elsewhere…)*<br>• [How do influences 'in the home' we talked about before affect these issues i.e. Access to basic needs, quality of relationships in the home, physical & mental health, 'personality'?]	*What are the effects of taking these steps?*

e) Prior experience with health research

Have you any direct experience of health research being conducted in the County that involved ALH? Please tell me more about that, including:

- The main aim of the study, who was conducting it, and what procedures were involved for the adolescents (particularly collection of samples e.g. blood, urine, stool etc)
- How consent/assent was obtained, including who from
- Do you think that participating in this study generated any challenges for the adolescents/anyone else? How do you think these challenges should/could be addressed? [Probe for any interactions with challenges already discussed – either made worse or mitigated by research]

At end of interview, ask for demographic information: education, age, religion

2. **TOOL FOR IDI STAFF/MATRON/OTHER WITH PARTICULAR RESPONSIBILITY TO SUPPORT STUDENTS WITH CHRONIC ILLNESSES IN SCHOOLS**

Introduction

Thank you for agreeing to talk to us today. We are working on a study that's looking at different aspects of the lives and experiences of adolescents in Kilifi, between the ages of around 13 to 18 years. Our study aims to feed into planning of future research that involves adolescents, to make sure that research plans take account of this group's particular

perspectives. In particular, we want to look for ways to ensure that the way that research in future does not cause any 'hidden' problems and does provide support to adolescents in areas where it can. Overall, we would like research plans to be more 'adolescent friendly'.

One particular area of interest in our study is understanding the everyday lives and experiences of adolescents who are living with chronic illnesses. We are particularly interested in learning about their experiences in school, so really appreciate learning more about this from you. We are interested in understanding what challenges they face at school in relation to their illness, any ways in which the school can support them, any strategies they use themselves, and how other students and teachers respond to these issues.

a) **Your roles & responsibilities**

- Can we start by learning more about the work that you do as a [staff member etc] in this school – what are your teaching and other roles in the school? How long have you worked in this position? Where were you working previously and what were your roles?
- Can you describe what involvement you have in supporting or working with students who have longstanding illnesses? (Probe for experiences with students with different conditions) Are there others in the school who have the same or other roles in working with this group of students? If so, what are these roles? How were you chosen to take on these roles? [Probe for own interest in doing this]
- **Managing students with chronic health conditions in schools** (e.g.HIV and SCD)*: Can you say something about these issues in this school, as well as in schools more generally*

In schools in this area, are there any guidelines/'policies'/agreed practices that address how students who have chronic health conditions should be managed or supported? For example…

- How do schools generally help students on long term medications to take these appropriately. How is the health of such students monitored?
- How is the decision on if a sick pupil should go home or stay in school made?
- What about support for students who need to go to clinics regularly – how is this managed in schools? How does the school help them compensate for the lost time in class?

- Does this vary depending on the type of illness they have? If so, can you say more about that? (Probe for HIV specifically)

b) **Relationships with other students and teachers**

- Do other teachers and students generally know about students living with longstanding illnesses?

Fellow students:

- How do other students generally interact with fellow-students who have longstanding illnesses? Are they generally treated the same way or differently to other boys and girls in schools?
- If differently, can you say more about how they would be treated differently – and what that depends on? (probe for type of illness/severity of illness, types of symptoms, personality issues, gender, age etc)
- Can you give any examples of situations you know about where a student has been isolated or rejected by their peers, because of their illness (in any school)? What was the illness/how were they isolated/what effect did this have? what was the outcome? How are such situations handled in schools?
- For example, we have been told that some students who are living with HIV are unwilling to disclose their status at school because they fear being stigmatized by other pupils. Do you think this is true – for example in this or other schools?
- Can you give any examples of students living with HIV who experienced stigma at (any) school that you could share to help us understand? What kind of stigma did they experience, how did they respond and what was the outcome?
- How typical was this student – do you think that many others have the same experience? (if not, why not)
- Do you have examples when students living with HIV were not stigmatized at school and were able to be open about their status with other students? Why do you think this student did not feel stigmatized, while others seem to find this very difficult? Is this a common situation, or unusual?

c) **Academic progress for students with chronic illnesses**

- How do students who have longstanding illnesses progress with their studies at school? Any differences compared to other students? What do you think brings about the differences? a [Probe for time off school, low concentration, mental health/social acceptance/exclusion by others at school, physical health etc.]
- Does the type of longstanding illness make a difference here? if so, in what way (probe for HIV specifically – whether and how this influences academic progress at school)
- What about the student's family support and home circumstances – how do you think this influence academic progress (if at all)?
- What changes could help them to make better academic progress?

d) **Emotional relationships**

As students reach adolescence, it is natural for them to start thinking about issues of 'boyfriends' and 'girlfriends' – which of course can make a teacher's life very complicated! Is this a concern among students and teachers in this school? Is it different for pupils with longstanding illnesses?

- What influences this normal part of development for this group? (Probe for type of illness, physical/mental health personality, family influences etc).

e) **Any experience with health research:** Have you any experience of health research being conducted in this school or other schools or involving children? If so:
    - What was the study about (aims), who was conducting it, and what did it involve for the students (e.g. answering questions, collection of samples e.g. blood, urine, stool etc)
    - How was consent/assent obtained, including who gave?
    - Do you think that participating in this study generated any challenges for students with chronic illnesses, particularly HIV? If so, how do you think these challenges should/could be addressed? [Probe for any interactions with challenges already discussed – either made worse or mitigated by research]

At end of interview, ask for demographic information: education, age, religion

3. **IN DEPTH INTERVIEWS WITH SOCIAL CARE PROVIDERS**

Introduction

Thank you for agreeing to talk to us today. We are working on a study that's looking at different aspects of the lives and experiences of adolescents in Kilifi, between the ages of around 13 to 18 years. Our study aims to feed into planning of future research that involves adolescents, to make sure that research plans take account of this group's particular perspectives.

In particular, we want to look for ways to ensure that the way that research in future does not cause any 'hidden' problems, and does provide support to adolescents in areas where it can. Overall we would like research plans to be more 'adolescent friendly'.

One particular area of interest in our study is understanding the everyday lives and experiences of adolescents living with HIV/AIDS (ALH), including those born with HIV and those who became infected later [refer to PMTCT if relevant].

We realise that you have a lot of experience in supporting families living with HIV, so really appreciate learning more about this from you. We understand that families' situations are very diverse, and that some will experience more challenges related to living with HIV/AIDS than others. We would like to learn about the range of experiences common in Kilifi, including for those living with HIV that find positive strategies to manage their situation, and others that find this more difficult.

During the interview, when we talk about 'issues' faced, we would like to understand what impact these issues have on people's lives, including over time. We would also like learn how adolescents and/or families tackle issues, and how well the approaches they use work. We understand that sometimes the way people tackle challenges might turn out to be very helpful but sometimes may not really help at all.

a) **Roles & responsibilities:** *Could we start by learning more about the work that you do as a [CHV etc]?*

- Can you describe your roles and responsibilities at the moment? [Probe to clarify if HIV/AIDS affected families only or wider responsibilities]
- [Where relevant] Roughly how many/what proportion of families in your care are living with HIV/AIDS vs not?

- Which organization(s) are you working with in this role? [will follow up more on this later Q x]
- Can you say something about how you were selected/recruited to work with this organization?
- How long have you been working in this way, and what were you doing before this?

b) **Social care/child services for adolescents/families living with HIV/AIDS:**
Numbers/patterns & referral pathways

- How many HIV affected children/adolescents/families are under your care?
- Which areas/sub locations in Kilifi do these families live in?
- How are children/families with HIV referred to you for support? [probe for circumstances or activities that lead to referral]
- How often do you visit/contact homes; and what mainly happens when you contact/visit? [if varies from visit to visit, try to understand what this pattern is]
- How do you interact/support children/families living with HIV that you visit?
    - exactly what support/services provided
    - how often visited
    - how long stay 'on books'
    - able to 'report back' & change according to need?

c) **Everyday lives of adolescents who are living with HIV/AIDs (ALH)**

Realising that the everyday experiences of adolescents and families who are living with HIV/AIDS (either infected or exposed uninfected adolescents) are likely to be very different, can we consider some specific aspects of their lives, and talk about the range of experiences you are aware of? These areas are: A. In the home; B. outside the home, including peer groups & schools; C. In accessing care. If you think of other areas, we will also include those. From time to time, I may ask you for specific examples of the issues you are describing.

Can you tell me something about the families/ALH that you are working with at the moment? What are the main kinds of challenges they face, including at home/in the family, at school or in the community and in accessing health care? I would like to hear about these challenges and the ways people handle them that you think are positive or

negative [Ask open question – listen to responses and p**robe** for following potential positive and negative influences on quality of life for ALH in less and more well-resourced families]:

<table>
<tr><th>Possible influences</th><th>Probes</th></tr>
<tr><td colspan="2">IN THE HOME</td></tr>
<tr><td>• Access to basic needs for family (shelter, food/nutrition, clear water, hygiene, menstruation for girls, economic status & changes in economic status e.g. from losing jobs or costs of accessing care)
• Nature and quality of relationships within the family (supportive/unsupportive) – parents, grandparents, siblings, extended family (positive and negative relationships: e.g. Loss of parents/abusive relationships/change of parents OR positive examples of living with HIV?
(May come up here or elsewhere…)
• Health/physical wellbeing (adolescent and others in family e.g. infection/stunting)
• Psychological wellbeing (adolescent and others in family e.g. depression, anxiety, loss of concentration?) - (probe for ‘acting out’ = high risk behaviour in relation to sexual activity, alcohol & drug use, other risks; and lack of progress at school)
• ‘Personality’ [Any external events affected these situations? E.g. Human rights issues, political instability, natural disasters, strikes, disease outbreaks]</td><td rowspan="3">Which challenges/issues are common/ important?

What are their impacts, and how does this differ between families?

What steps do ALH/families take to manage these issues?</td></tr>
<tr><td>OUTSIDE THE HOME: SCHOOL/OTHER SOCIAL GROUPS</td></tr>
<tr><td>• Nature and quality of relationships outside home (supportive/unsupportive including STIGMA):
o in school - friends and teachers/school nurses etc
o outside school – other peer groups e.g. church youth groups, sports clubs etc
o boyfriend/girlfriend relationships
o social media</td></tr>
</table>

• At school/access to learning: attendance, concentration, progress and inclusion at school<br>*(May come up here or elsewhere…)*<br>• [How do influences 'in the home' we talked about before affect these issues i.e. Access to basic needs, quality of relationships in the home, physical & mental health, 'personality'?]	*What are the effects of taking these steps?*
**IN ACCESSING HEALTH CARE**	
• Which governmental and NGO services are focused on children ALH in Kilifi? [*Any documentation we could look at?*] (e.g. Afya Pwani, Plan etc) How do they work/contribute?<br>• Could you describe which organisations you are connected to, and in what way? [Probe for NGO & CBO & MoH etc, be clear about main reporting lines e.g. Nilinde/CBO/KCR etc<br>• Given all the issues you mentioned (Q5), how well are these services working to help with these challenges?<br>• In your opinion, what are the most important ways your services should be supported or strengthened/expanded, and why? What resources would this need?	

4. **GROUP INTERVIEW WITH KENEPOTE TEACHERS**

Introduction:

We are working on a study that's looking at different aspects of the lives and experiences of ALHIV in Kilifi, between the ages of around 13 to 18 years. Our study aims to feed into planning of future research that involves this & other groups of adolescents, to make sure that research plans take account of this group's particular perspectives. In particular, we want to look for ways to ensure that the way that research in future does not cause any 'hidden' problems, and does provide support to adolescents in areas where it can. Overall we would like research plans to be more 'adolescent friendly'.

a) **KENEPOTE** as an organization – how/when set up? Main aims/ways of working? Any reference materials?

b) **Roles & responsibilities & schools policies for ALHIV**

- What teaching/other roles in school? Specifically for ALHIV in schools?
- What guidelines/'policies'/agreed practices to support ALHIV? How does these work out in practice?

  For example…

  - Helping students take long term medications appropriately or attend clinics regularly?
  - Managing/monitoring physical or mental health?

- How is HIV covered in the school curriculum?
- Any differences in policies or practices for HIV and other chronic health conditions?

c) **Relationships with other students and teachers**

Fellow students:

- How do ALHIV generally interact with fellow-students?
- Any examples of **positive** and **negative** interactions – what issue, how did they respond, with what outcome?
- Why might some ALHIV experience more problems with relationships than others? What other factors?

Teachers:

- How do teachers in general (in your school and in other schools) respond to ALHIV in their classes?
- Can you give any specific **positive** and **negative** examples of student-teacher relations – e.g. what exactly did they do to support/not support? And what was the effect of that?

Across all these areas – policies & support in schools, peer and teacher relations:

- Do you think there are differences for ALHIV who are vertically vs horizontally infected?
- Do you have any recommendations on ways school policies/practices could be strengthened for ALHIV?

d) **Involving ALHIV in health research** [Explain why research involving ALHIV is important & sensitive]

- Could you say something about how reasonable or important you think it is to involve ALHIV in research?
  - What kind of research would it be **reasonable**/**not reasonable** to involve them in, and why? (prompt –questionnaire study, research involving taking samples, behavioral intervention trial etc)

Explain AHOS, including run with support of CDE/TSC. At clinic, range of cognitive functioning test (pattern recognition, maths, comprehension etc), clinical examination and computerised self-administered questionnaire risk taking behaviour. Each visit lasts about 3 hours. CDE given letter for time off school to attend.

Given that researchers want to make sure that AHOS is as ALHIV-friendly as possible, what would be the most important considerations for researchers planning to invite ALHIV to join AHOS?

- o *About timing? About consent/assent processes? About avoiding stigma/disclosure? Anything else?*

5. GROUP INTERVIEW WITH HIV HEALTH CHAMPIONS

Introduction: Thank you for agreeing to talk to us today. We are working on a study that's looking at different aspects of the lives and experiences of adolescents in Kilifi, between the ages of around 13 to 18 years. Our study aims to feed into planning of future research that involves adolescents, to make sure that research plans take account of this group's particular perspectives.

In particular, we want to look for ways to ensure that the way that research in future does not cause any 'hidden' problems and does provide support to adolescents in areas where it can. Overall, we would like research plans to be more 'adolescent friendly'.

One particular area of interest in our study is understanding the everyday lives and experiences of adolescents living with HIV/AIDS (ALH), including those born with HIV and those who became infected later [refer to PMTCT if relevant].

We realise that you have a lot of experience in supporting peers living with HIV, so really appreciate learning more about this from you. We understand that families' situations are very diverse, and that some will experience more challenges related to living with HIV/AIDS than others. We would like to learn about the range of experiences common in Kilifi, including for those living with HIV that find positive strategies to manage their situation, and others that find this more difficult.

During the interview, when we talk about 'issues' faced, we would like to understand what impact these issues have on people's lives, including over time. We would also like learn how adolescents and/or families tackle issues, and how well the approaches they use work. We understand that sometimes the way people tackle challenges might turn out to be very helpful but sometimes may not really help at all.

a) **Could you share some information about yourself?**

- Favorite subject?
- Hobby? What do you want to be when you grow up?
- Your friends? in school? At home?
- How chosen as ALHIV champion? Trainings attended?
- Roles as a champion? What activities, when, where.

b) **Can you tell me something about the families/ALH that you know about at the moment? What are the main kinds of challenges they face, including <u>at home/in the family</u>, <u>at school or in the community</u> and in <u>accessing health care</u>? I would like to hear about these challenges and the ways people handle them that you think are positive or negative** [Ask open question – listen to responses and p**robe** for following potential <u>positive</u> and <u>negative</u> influences on quality of life for ALH in less and more well-resourced families]:

Possible influences	***Probes***
**IN THE HOME**	

<table>
<tr>
<td>

- Access to **basic needs** for family (shelter, food/nutrition, clear water, hygiene, menstruation for girls, economic status & changes in economic status)
- Nature and quality of **relationships** within the family (supportive/unsupportive) – parents, grandparents, siblings, extended family (positive and negative relationships:

(May come up here or elsewhere…)

- Health/physical wellbeing (adolescent and others in family e.g. infection/stunting)
- Psychological wellbeing (adolescent and others in family e.g. depression, anxiety, loss of concentration*? (alcohol and substance abuse)*
- ‘Personality’ [Any external events affected these situations? E.g. Human rights issues, political instability, natural disasters, strikes, disease outbreaks]

</td>
<td rowspan="3">

Which challenges/ issues are common/ important?

What are their impacts, and how does this differ between families?

What steps do ALH/families take to manage

</td>
</tr>
<tr>
<td>

OUTSIDE THE HOME: SCHOOL/OTHER SOCIAL GROUPS

</td>
</tr>
<tr>
<td>

- Nature and quality of relationships outside home (supportive/unsupportive including STIGMA):
    - in school - friends and teachers/school nurses etc
    - outside school – other peer groups e.g. church youth groups, sports clubs etc
    - boyfriend/girlfriend relationships
- social media
- At school/access to learning: attendance, concentration, progress and inclusion at school
- What happens when ALH have to miss school to attend their clinic visits?
- How do the ALHIV get the permission to attend clinic if their status is not known at school? Who is best placed to know about ALHIV at school and why?
- [How do influences ‘in the home’ we talked about before affect these issues i.e. Access to basic needs, quality of relationships in the home, physical & mental health, ‘personality’?]

</td>
</tr>
</table>

IN ACCESSING HEALTH CARE	*these*
• Where do most ALHIV that you know of attend their clinics? Cost of accessing<br>• health care? Distance they have to travel to go to the clinic?<br>• What do they like about the clinics?<br>• What do they dislike?<br>• What happens when ALH miss clinic visits because of commitments at school or elsewhere?<br>• What can be done to improve on the health services?<br>• Tell me about adherence to medication amongst your peers? How does the clinic/school/ home environment affect adherence? What do ALH do to counter the challenges?<br>• What support systems are available to support ALHIV with adherence to drugs? At CCC, support groups, mentor mothers, to ensure drugs adherence?<br>• Do you have any positive stories you have experienced and which you can share with us in living with HIV and accessing care?<br>• Which governmental and NGO services are focused on children ALHIV and their families in Kilifi?] (e.g.Nilinde, compassion) How do they work/contribute?<br>• Tell me about support groups for ALHIV? How often they meet, activities they engage in? who leads? What do you feel about the groups? What could be done to make them more productive?	*issues?*<br><br>*What are the effects of taking these steps?*

c) **Experience with health research**

- Tell me about the study that you joined in KEMRI? (can mention the one they play computer games) any other study, how different was it from this one……….
- How were you informed about the study? Why did you agree to join?
- Now tell me about your participation in that study? What happens when you come for the visit?

- What do others who come to the study say about the study? How about those who did not join the study?
- What do you like about coming for the study visits?
- What would you want changed about the study? Probe on disliked procedures and activities and how these could be changed.
- How can future studies be designed to take care of your needs/suit you/be youth friendly?

6. **FGD YOUNG PEOPLE LIVING WITH HIV/AIDS**

Introduction

Thanks for coming to the recent KEMRI workshop to talk about experiences of being in KEMRI research (AHOS) – we've invited you here today to talk more about some of the issues that came up at the workshop, and others that have come up in conversations we've had with others people as part of our research.

Today we'd like to ask you to talk more about some of the areas we talked about at the workshop. One area is about your experiences of participating in the KEMRI study at the NA clinic (AHOS). Then we would also like to learn more about typical life experiences for ALHIVs in general - based on what you know e.g. as an adherence counsellor or just as a friend. In terms of life experiences, we are not asking you to share your own personal experiences – unless you think this is very relevant and would like to do that.

Overall, we are keen to learn about positive experiences and challenges that ALHIV commonly encounter in their normal lives – including at home and at school – as well as any ideas you have about how things could change to make life easier for ALHIV.

……

RESEARCH

a) Thinking back to when you first learned about the KEMRI study being run here at the NA clinic:

- Can you explain more about how you and your parents learned about the study? (Probe on who gave information, to whom and when/where – how the teenager & parents talked about this, together or separately)
- How did you make the decision to join (probe on roles of teenager and parents, or any other person (e.g. FW) and whether/how discussed together as family)?
- What made you decide to join the study – what did you think the main point of the study was? (probe – to see if seen as benefitting themselves in some way, or having some other wider/longer term benefit for others *who were not in the study* will benefit – i.e. research)
- Have your views on the KEMRI study changed over time at all? In what way? (probe for changes in perceptions of the study aims AND in attitudes to study)

b) At the workshop, we learned that some students had difficulties in getting time off school for the research visits to KEMRI.

- Can you say more about these potential challenges – what are the main challenges experienced, and how commonly do they happen?
- How do you think they should be addressed?

[*If not brought up – prompt for…*] The difficulties we talked about were that:

- it was hard to catch up on lessons missed later so risked getting behind with work
- it was necessary to explain to a teacher why missing school (to participate in the research) and this risked disclosing HIV status given topic (link to discussion above about stigma in schools, if described)

HOME

c) Many people have talked about how important the **home environment** is for ALHIV's wellbeing generally (e.g. being able to manage their lives outside the home well). Could you say what you think about that view - from your experience of talking to many ALHIVs?

[*If agree*] Can you say more about HOW you think the home environment is important for ALHIV's wellbeing? [*Probe for ways home may be supportive and/or create challenges, including emotional & practical support e.g. taking ARTs together, choosing right schools, providing safe & nurturing environment – food, social, emotional]*

- Can you give any specific examples where the home environment was helpful? What exactly was helpful, in what way? [*Probe for roles of particular individuals in family and details of how support/lack of support worked out*]
- What about situations where the home environment was not helpful? *[Probe as above*]
- How common are these (positive and negative) experiences amongst ALHIV in your experience?
- Do you think that there have been changes in relation to nature of these issues or how commonly they happen over the past few years – if so, what makes you think that?

[*if don't agree*] Can you say more about why you don't think the home environment is important to ALHIV's wellbeing? *[Probe for views that other areas of life are more important – and how/why]*

SCHOOL

d) What about if we turn to the school environment? We've learned that for many ALHIV, school is also a place that's important to ALHIV's wellbeing. Could you say what you think about that view?

[*If agree*] Can you say more about HOW you think the school environment is important for ALHIV's wellbeing? [*Probe for ways school may be supportive and/or create challenges, particularly around ability to form friendship groups, risks of inadvertent disclosure and stigma/discrimination and educational progress at school]*

- Can you give any specific examples where the school environment was helpful? What happened, in what way?
- What about situations where students met challenges at school? What happened, in what way?
- How common are these (positive and negative) experiences amongst ALHIV in your experience?
- Do you think that there have been changes in relation to nature of these issues or how commonly they happen over the past few years – if so, why do you think that?

[*If don’t agree*] Can you say more about why you don’t think the school environment is important to ALHIV’s wellbeing? *[Probe for views that other areas of life are more important – and how/why]*

e) Can you think of any ways in which these issues that ALHIV commonly encounter in schools should be addressed? Please explain more about that, and what it would involve.

7. **FGD WITH CAREGIVERS OF YOUNG PEOPLE LIVING WITH HIV/AIDS**

Introduction

Thanks for agreeing for your teenager to participate in the recent KEMRI workshop to talk about experiences of being in KEMRI research (AHOS) – we’ve invited you here today to talk more about some of the issues that came up at the workshop, and others that have come up in conversations we’ve had with others people as part of our research. Today we’d like to ask you about your experiences of the KEMRI study being run here at the NA clinic. We’d also like to talk to you about typical life experiences for many families living with ALHIVs in general but we are not asking you to share your own personal experiences – unless you think this is very relevant and would like to do that. Overall, we are keen to learn about positive experiences and challenges that ALHIV commonly encounter in their normal lives – including at home and at school – as well as any ideas you have about how things could change to make life easier for ALHIV.

………

RESEARCH

a) Thinking back to when you first learned about the KEMRI study being run here at the NA clinic:
    - Can you explain more about how you and your child learned about the study? (Probe on who gave information, to whom and when/where how the parents and child talked about this together or separately)

- How did you make the decision to join – who was the main decision maker? (Probe on roles of child and parents, or any other person (e.g. FW) and whether/how discussed together as family)?
- In general, what role do you think teenagers like your child should have in making this decision? What are the reasons you feel this?
- What made you decide to join the study – what did you think the main point of the study was? (Probe – to see if seen as benefitting their child in some way, or having some other wider/longer term benefit for others *who were not in the study* will benefit – i.e. research)
- Have your views on the KEMRI study changed over time at all? In what way? (probe for changes in perceptions of the study aims AND in attitudes to study)

HOME

b) Many people have talked about how important the **home environment** is for ALHIV's wellbeing generally (e.g. being able to manage their lives outside the home well). Could you say what you think about that view based on what you know about families in this situation who are living in this area?

[*If agree*] Can you say more about HOW you think the home environment is important for ALHIV's wellbeing? [*Probe for ways home may be supportive and/or create challenges, including emotional & practical support e.g. taking ARTs together, choosing right schools, providing safe & nurturing environment – food, social, emotional]*

- Can you give any specific examples where the home environment has been helpful? What exactly was helpful, in what way? [*Probe for roles of particular individuals in family and details of how support/lack of support worked out*]
- What about situations where the home environment was not helpful? *[Probe as above*]
- How common are these (positive and negative) experiences amongst ALHIV in your experience?
- Do you think that there have been changes in relation to nature of these issues or how commonly they happen over the past few years – if so, what makes you think that?

[*if don't agree*] Can you say more about why you don't think the home environment is important to ALHIV's wellbeing? *[Probe for views that other areas of life are more important – and how/why]*

SCHOOL

c) What about if we turn to the school environment? We've learned that for many ALHIV, school is also a place that's important to ALHIV's wellbeing. Could you say what you think about that view?

[*If agree*] Can you say more about HOW you think the school environment is important for ALHIV's wellbeing? [*Probe for ways school may be supportive and/or create challenges, particularly around ability to form friendship groups, risks of inadvertent disclosure and stigma/discrimination and educational progress at school]*

- Can you give any specific examples where the school environment was helpful? What happened, in what way helpful?
- What about situations where students met challenges at school? What happened, in what way?
- How common are these (positive and negative) experiences amongst ALHIV in your experience?
- Do you think that there have been changes in relation to nature of these issues or how commonly they happen over the past few years – if so, what makes you think that?
- [*If don't agree*] Can you say more about why you don't think the school environment is important to ALHIV's wellbeing? *[Probe for views that other areas of life are more important – and how/why]*

d) Can you think of any ways in which issues that ALHIV commonly encounter in schools should be addressed? Please explain more about that, and what it would involve.

e) Can we talk about the process of your teenager getting time off school to take part in the KEMRI study at the NA clinic? In your experience, how <u>easy</u> or <u>difficult</u> has this been?
    - [For any challenges mentioned] Can you say more about these potential challenges – what are the main challenges experienced, and how commonly do they happen?

What did you or your child do to get round these challenges, and how did that work out?

- Do you have any recommendations for KEMRI or schools on ways these particular challenges should be avoided?
